# Extracellular Vesicles from Adipose Tissue-Derived Stromal Cells Stimulate Angiogenesis in a Scaffold-Dependent Fashion

**DOI:** 10.1007/s13770-024-00650-4

**Published:** 2024-07-08

**Authors:** V. E. Getova, E. Orozco-García, S. Palmers, G. Krenning, R. Narvaez-Sanchez, M. C. Harmsen

**Affiliations:** 1 Department of Pathology and Medical Biology, University Medical Center Groningen, University of Groningen, Hanzeplein 1 (EA11), 9713 GZ Groningen, The Netherlands; 2 University Medical Center Groningen, W.J. Kolff Research Institute, University of Groningen, Groningen, The Netherlands; 3 Department of Clinical Pharmacy and Pharmacology, University Medical Center Groningen, University of Groningen, Groningen, The Netherlands; 4 Physiology and Biochemistry Research Group–PHYSIS, Faculty of Medicine, University of Antioquia, Medellin, Colombia

**Keywords:** Extracellular vesicles, EV, ASC, Angiogenesis, Vascularisation, Cell communication, miRNA

## Abstract

**Background:**

The extracellular vesicles (EVs) secreted by adipose tissue-derived stromal cells (ASC) are microenvironment modulators in tissue regeneration by releasing their molecular cargo, including miRNAs. However, the influence of ASC-derived extracellular vesicles (ASC-EVs) on endothelial cells (ECs) and vascularisation is poorly understood. The present study aimed to determine the pro-angiogenic effects of ASC-EVs and explore their miRNA profile.

**Methods:**

EVs were isolated from normoxic and hypoxic cultured ASC conditioned culture medium. The miRNA expression profile was determined by miRseq, and EV markers were determined by Western blot and immunofluorescence staining. The uptake dynamics of fluorescently labelled EVs were monitored for 24 h. ASC-EVs' pro-angiogenic effect was assessed by sprouting ex vivo rat aorta rings in left ventricular-decellularized extracellular matrix (LV dECM) hydrogel or basement membrane hydrogel (Geltrex®).

**Results:**

ASC-EVs augmented vascular network formation by aorta rings. The vascular network topology and stability were influenced in a hydrogel scaffold-dependent fashion. The ASC-EVs were enriched for several miRNA families/clusters, including Let-7 and miR-23/27/24. The miRNA-1290 was the highest enriched non-clustered miRNA, accounting for almost 20% of all reads in hypoxia EVs.

**Conclusion:**

Our study revealed that ASC-EVs augment in vitro and ex vivo vascularisation, likely due to the enriched pro-angiogenic miRNAs in EVs, particularly miR-1290. Our results show promise for regenerative and revascularisation therapies based on ASC-EV-loaded ECM hydrogels.

**Supplementary Information:**

The online version contains supplementary material available at 10.1007/s13770-024-00650-4.

## Introduction

Adipose tissue-derived stromal cells (ASC) are a subcategory of white fat-derived mesenchymal stromal cells (MSC) [1] that have been in the spotlight of regenerative medicine during the last few years. ASC are multipotent plastic adherent highly proliferative cells [2] that express pericyte markers and stimulate vascularisation in vitro and clinically [3–5]. While ASC secrete regenerative factors such as HGF, VEGF, and FGF2, these have been administered locally in clinical trials or systemic in animal trials to promote wound healing, including extracellular matrix (ECM) remodelling, vascularisation, and counteract fibrosis [6–9]. A remaining challenge in clinical application is that both ASC and their secreted trophic factors appear to rapidly disappear from the injection site due to diffusion or cellular migration. Yet, clinical efficacy endures that ASC, ASC-derived extracellular vesicles (ASC-EVs), and ASC-conditioned media (ASC-CM) are beneficial in treating myocardial infarction, kidney injury, bone regeneration, graft rejection, and autoimmune diseases [4, 5].

A remaining challenge in clinical application is that both ASC and their secreted trophic factors appear to rapidly disappear from the injection site due to diffusion or cellular migration. For this reason, other nature-inspired therapeutic modalities with better retention in lesions and tissues are warranted. In normal physiology, most cells produce and secrete lipid membrane-shelled extracellular vesicles (EVs). These EVs may travel over large distances via peripheral circulation or over short distances in tissues. EVs are present in all biological fluids, including blood, urine, and saliva, as well as in cell culture-conditioned mediums. The cargo of EVs has a diverse molecular makeup that ranges from proteins and lipids to nucleic acids, which all affect the function of cells' uptake of EVs. Therefore, EVs are a regulatory system beside the long-studied canonical paracrine and juxtacrine signalling pathways.

MicroRNAs are the best-studied cargo of EVs and may contribute to vascularisation [10]. Vascularisation is hypoxia-driven, and we showed that the ASC’s secretome is indeed affected by hypoxic culturing [11]. Previously, we showed that molecular secretome components of ASC’s conditioned media (ASC-CM) are efficiently bound and constantly released by organ-derived extracellular matrix (ECM) hydrogels [12, 13]. We also showed that 3D systems of these hydrogels support the vascularisation of microvascular endothelial cells [14]. Therefore, we reasoned that hypoxia promotes the pro-angiogenic capacity of ASC-derived extracellular vesicles (ASC-EVs) by upregulating pro-angiogenic molecules, aka angio-miRNAs [15]. In contrast, we further reasoned that ECM-derived hydrogels are efficient platforms for delivering EVs over more extended periods.

This study contributes to understanding the role of ASC-EVs as potential cell-free angiogenic therapy for tissue repair and regeneration. It also demonstrates their efficiency in combination with ECM-derived hydrogels, which can improve retention and transient molecular release in future therapeutic approaches.

## Materials and methods

### Adipose stromal cell isolation and culture

ASC were isolated from subcutaneous adipose tissue obtained by liposuction from healthy human donors as previously described and characterized [12]. The ASC were cultured in Dulbecco's Modified Eagle Medium (DMEM; 1 g/L glucose, Gibco, Life Technologies, USA) supplemented with 10% Foetal Bovine Serum (FBS; Gibco, Life Technologies), 1 mM L-glutamine (Sigma-Aldrich, USA) and 0.1% Bovine Serum Albumin (BSA, A8806-5 g, Sigma, St Louis, United States), 100 U/mL penicillin/streptomycin (Sigma-Aldrich, USA) at 37 °C in a humidified atmosphere with 95% air and 5% CO_2_. Maximum passage five was used. The ASC from these batches were previously characterized and confirmed by FACS analyses for positive and negative surface markers, including CD45, CD31, CD29, CD90, and CD105 [16]. To compensate for donor heterogeneity, ASC were cultured as pools of five donors, all combined and consistently used in this fashion for all experiments.

### ECs culture

Human umbilical vein endothelial cells (HUVECs) were obtained from the Endothelial Cell Facility (University Medical Center Groningen, Groningen, The Netherlands) and cultured on gelatine–precoated tissue culture flasks (Nunc ™ EasYFlask ™ Thermo Fisher, The Netherlands) in Endothelial Cells Culture Medium (ECM), RPMI (Lonza, Switzerland) supplemented with 20% FBS (Gibco, Life Technologies), 50 µg/mL crude ECGF solution, 2 mmol/L L-Glutamine (Sigma-Aldrich, St. Louis, MO, USA), 5 U/mL Heparin, 100 U/ml Penicillin, 100 µg/mL Streptomycin (Sigma-Aldrich, St. Louis, MO, USA) at 37C°, 5% CO_2_ HUVEC (max passage 5) were harvested by trypsinization (0.5% trypsin–EDTA, Sigma-Aldrich) at 80% confluence. Only *Mycoplasma spp*. free cells were used in these experiments.

### Conditioned medium (CM) and EV isolation

**Fig. 1:**
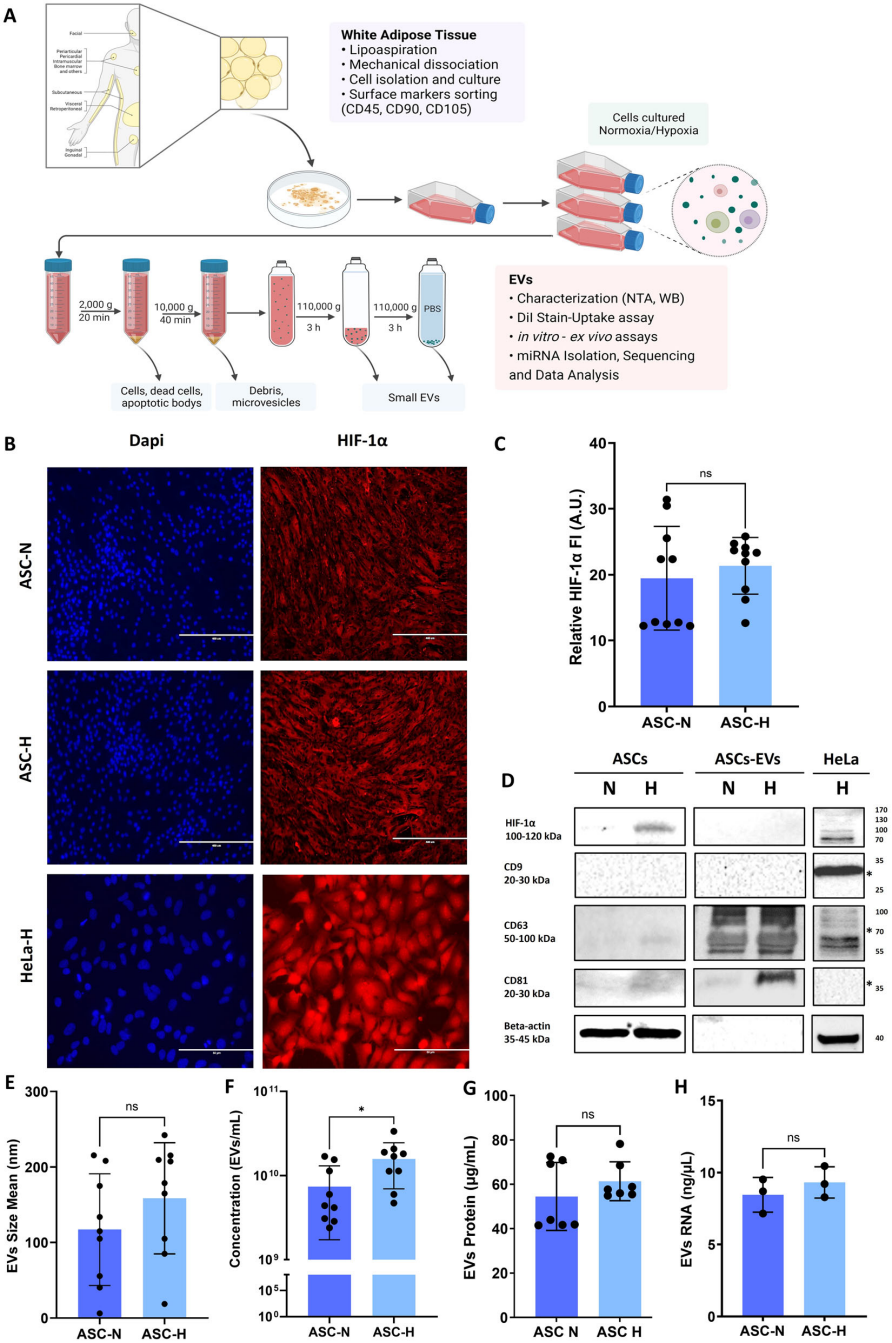
Characterisation of ASCs and ASCs-derived EVs in normoxia and hypoxia. **A** Workflow for EV isolation and procedures**.** The ASC-EVs production, isolation, characterisation, and bioassays in ECs and aorta segments. *Made in biorender*. **B** Immunofluorescence of ASC cultured in normoxia (N), hypoxia (H), and HeLa cells, stained for Hypoxia Inducible Factor (HIF-1*α*). HIF-1*α* was detected with Rabbit HIF-1*α*, and Alexa Fluor 594 anti-Rabbit nuclei were stained with Dapi. Scale bar 400 µm and 50 µm. **C** Hypoxia did not increase the accumulation of HIF-1*α* in ASC. Image quantification HIF-1*α* fluorescence intensity (FU) in arbitrary units for the stain control. **D** The expression of HIF-1*α* and EV-enriched proteins, including CD9 (no detected), CD63, and CD81 in ASC and ASC-derived EVs, was confirmed by western blot. HeLa cells cultured in hypoxia were used as control, * corresponding to EVs derived from HeLa hypoxia cells. **E** The mean size and **F** particle concentration of the ASCs-derived EVs were identified by NTA. Hypoxia did not alter **G** protein and **H** RNA concentration. Mean ± SD error bars were calculated based on at least three independent experiments (n = 3) performed in triplicates. Multiple comparisons *p* value < 0.05 (*); ns: not significant. Each point in the graphs represents an individual sample.

Subconfluent ASC in 175 cm^2^ flasks were washed twice with Dulbecco’s phosphate-buffered saline (DPBS; BioWhittaker®, Walkersville, MD, USA). Cells were incubated in normoxia (N, 21% oxygen) and hypoxia (H, 1% oxygen) for 24 h in a serum-free medium (DMEM, Gibco, Life Technologies, USA). Next, the ASC-conditioned media (ASC-CM) was collected and concentrated 20-fold with 3 kDa MW cutoff Amicon filters (Sigma Aldrich UFC900308). The EVs were isolated by differential centrifugation according to Théry et al. [17] with minor modifications (Fig. 1A). Briefly, the medium was collected and centrifuged at 2,000 xg for 20 min to remove intact detached cells and debris. The supernatant was centrifuged at 10,000 xg for 40 min to remove big-sized EVs and other debris. Subsequently, the supernatant was ultracentrifuged at 110,000 xg for 3 h. The pellet containing the EVs was collected and washed with PBS and centrifuged again at 110,000 xg for 3 h. All centrifugation steps were performed at 4 °C. Ultracentrifugation was performed with a Type 45Ti rotor (K factor:133, #339,160, Beckman Coulter®, USA) with polycarbonate bottles (#355,622, Beckman Coulter®, USA) and a SW55Ti rotor (K factor:48, #342,196, Beckman Coulter®, USA) with tubes (#326,819, Beckman Coulter®, USA), with maximum acceleration and deceleration. The EV samples were aliquoted and stored at − 80 °C until further use. Every batch of CM or EVs is obtained from a batch of ACS cell culture. The supernatants of 15 T75 cell culture bottles, with 3 replicates. All EV batches were obtained from cells in passages 4–5.

### Decellularized extracellular matrix (dECM) hydrogel

The decellularized extracellular matrix of the left ventricle (LV dECM) was produced from healthy porcine hearts purchased from a local slaughterhouse (Kroon Vlees, Groningen, the Netherlands). The left ventricle was cut into small pieces (1-2mm^3^), then mechanically minced in a commercial kitchen blender (Bourgini 21,300, Breda, The Netherlands) to a homogeneous paste, which was digested with 0.05% trypsin (Thermo Fisher Scientific, Waltham, MA, USA) for 3 h. Afterwards, it was incubated with excess NaCl-saturated solution for 3 h. Next, the crude LV dECM extract was incubated for 24 h with 1% SDS (Sigma-Aldrich). Followed by 24 h incubation with 1% Triton X-100 solution (Sigma-Aldrich). The next day, the material was incubated for 24 h with 1% sodium deoxycholate (Sigma-Aldrich), followed by 24 h incubation with DNase solution (DNase, 30 µg/mL in 1.3 mM MgSO4 and 2 mM CaCl; Roche Diagnostics GmbH Mannheim, Germany). The extract was washed with 96% ethanol for 3 h. All previous steps were performed at 37C° with constant shaking; samples were extensively washed multiple times with sterile PBS between steps. Finally, the LV dECM was freeze-dried (Labconco, Kansas City, MO, USA) overnight and then ground into a powder with an ULTRA-TURRAX (IKA, Staufen, Germany). To produce the hydrogel, the LV dECM was digested with porcine pepsin (2 mg/mL, 3200 I.U., Sigma-Aldrich, St. Louis, MO, USA) in 0.01 M hydrochloric acid (HCl) for 6 h, at room temperature (RT) and constant stirring. Then, the pH was neutralized by adding 1/10th volume of 0.1 M sodium hydroxide (NaOH) and 1/10th volume of 10 × PBS to generate a neutral and isotonic hydrogel. Hydrogel has a final concentration of 10 mg/ml of LV dECM and was stored at 4 °C.

### Nanoparticle tracking analysis (NTA)

The particle size distribution and concentration of EVs were determined by NTA equipped with a 405 nm laser with an LM14 module and the NTA software (Ver. 3.0.) to track each particle on a frame-by-frame basis (NanoSight NTA 3.0, Malvern Instruments, Amesbury, UK) used according to the manufacturer’s recommendations. The EVs were diluted tenfold and 100-fold in double-filtered Dulbecco’s phosphate-buffered saline (DPBS; BioWhittaker®, Walkersville, MD, USA) before analysis, and sole DPBS was used as control. The analysis settings were optimised, and the individual size distribution of five captures (60 s each) per sample was repeated 5 times with a minimum of 50 particles per frame. The data were analysed to obtain the mean, mode, median, and estimated concentration of each particle size per image.

### Protein isolation and quantification

Cells and EVs were lysed in RIPA buffer (Pierce, Thermo Fisher Scientific Inc, Netherlands), supplemented with Protease (Sigma-Aldrich, P8340, 1:100 v/v) and Phosphatase (Thermo Fisher Scientific, 78,420, 1:250 v/v) inhibitor cocktails. The protein concentration of the cell lysates was determined using the BCA Protein assay kit (Thermo Fisher Scientific), while the protein concentration in conditioned medium and isolated EV samples was quantified using the micro-BCA Protein Assay Kit (Thermo Fisher Scientific) according to the manufacturer’s instructions. The absorbance was read at 562 nm with Benchmark Plus™ microplate spectrophotometer system (Bio-Rad, Hercules, CA, USA). The protein concentration (µg/mL) was determined based on a calibration curve from a dilution series of bovine serum albumin (BSA, Thermo Fisher Scientific). DPBS served as a blank. n = 3 in triplicate.

### Western blot (WB) analysis

A total of 20–40 μg of isolated protein from both cell extract and EVs was separated by sodium dodecyl sulfate–polyacrylamide gel electrophoresis (SDS-PAGE) and transferred onto polyvinylidene fluoride (PVDF) membranes (Immobilon®-FL, Millipore, Germany) using a Trans-Blot Turbo Transfer System (Bio-Rad). The membranes were blocked for 1 h in 5% non-fat dry milk powder in TBST buffer (0.1% Tween 20 in Tris-PBS buffer). Then, the membranes were incubated overnight at 4 °C with the primary antibody. Primary antibodies used: anti-CD63 (1:1,000; ab68418, Abcam, UK), anti-CD81(1:800; ab109201, Abcam), rabbit anti-CD9 (1:1,000; ab92726, Abcam), mouse anti-GAPDH antibody (2118, Cell Signaling Technology; CST), rabbit anti-β-actin primary antibody (1:1,000, Cell Signaling; 4967L), and rabbit HIF-1*α* (1:1,000; 737R, Bioss). Next, membranes were washed in Tris-buffered saline (pH 7.4) and incubated for 1 h at RT with horseradish peroxidase (HRP)-conjugated donkey anti-mouse secondary antibody (1:5,000; 6410–05, Southern Biotech) or HRP-conjugated goat anti-rabbit secondary antibody (1:5,000; 4050–05, Southern Biotech). Immobilon® Forte Western HRP Substrate (Sigma-Aldrich) was used to visualise the chemiluminescent protein bands after exposure with ChemiDoc XRS + System (Bio-Rad). ImageJ software (Wayne Rasband NIH-USA v1.51 A, available at http:// rsb.info.nih.gov./ij/) was used to quantify the measured signals.

### EV labelling

Isolated EVs derived from ASC cultured in normoxia and hypoxia were labelled with the lipophilic dye DiI (D3911, ThermoFisher Scientific, Life Technologies, USA) according to the manufacturer’s instructions. Briefly, the EVs were incubated with 1 µM DiI Cell-Labelling Solution at 37 °C for 1 h with gentle agitation and then were washed twice with filtered DPBS and ultracentrifuged at 110,000xg for 3 h at 4 °C to ensure removal of DiI excess. A DiI dye background control sample was prepared following the above procedures but without using any ASC-EVs. The stained EVs were quantified by NTA.

### EV uptake

HUVEC cells were seeded into 12-well plates, 20,000 cells/cm^2^. After 24 h, a cold medium with DiI-stained ASC-EVs (1 × 10^3^ EVs/cell) or without EVs (control) was added for 1 h at 4 °C. Cells were transferred to 37 °C, and uptake was determined after 0, 3, 6, 12, and 24 h incubation, using flow cytometry and fluorescence microscopy. Flow cytometry cells were washed twice with DPBS, trypsinised, and washed twice in DPBS supplemented with 5% FBS to remove extracellular EV. Flow cytometry was performed on a NovoCyte Quanteon System (Agilent, USA) with default gain control settings. Autofluorescence was compensated for gating the cell population above the 5% threshold. Forward scatter (FSC-HLog) versus red fluorescence (RED-HLog) were recorded and analysed using FlowJo software. For fluorescent microscopy, cells were washed three times with DPBS, fixed with 4% paraformaldehyde (PFA), and incubated with 4',6-Diamidino-2-phenylindole (DAPI, 1 µg/mL, D9564, Sigma) and phalloidin coupled to Alexa Fluor®488 (1:500, Life Technologies) for 1 h at 37 °C. Images were acquired with a Leica fluorescence microscope (DM4000B, Leica Microsystems, Wetzlar, Germany). Image analyses and quantifications were made using the ImageJ software (*Wayne Rasband NIH-USA v1.51 A, available at *http:// rsb.info.nih.gov./ij/). Data from three independent experiments were used to generate results.

### Immunofluorescence

ASC (normoxic and hypoxic pre-conditioned) and HeLa, as control, were fixed with 4% PFA and incubated for 10 min with 50 mM NH_4_Cl. After permeabilisation (0.3% Triton-X for 5 min) and blocking (PBS with 5% donkey serum, Agilent Technologies; and 1% BSA, Sanquin) for 1 h, the antigens were probed with a rabbit anti-HIF-1*α* antibody (1:300; 0737R, Bioss), revealed with an Alexa Fluor 594-coupled donkey-anti rabbit antibody (1:500; A21207, Life Technologies). Nuclei were stained with DAPI (1 µg/mL, D9564, Sigma) and actin with Alexa-Fluor 488-phalloidin. Images were acquired with a Leica fluorescence microscope (DM4000B, Leica Microsystems, Wetzlar, Germany). Micrographs were analysed using ImageJ software (Wayne Rasband NIH-USA v1.51 A, available at http:// rsb.info.nih.gov./ij/. n = 3 in triplicate, a minimum of 3 random fields per replicate were quantified. The mean fluorescence intensity per cell was calculated.

### Aorta ring sprouting assay

Aortas from male Hsd: Sprague Dawley® SD® rats (Envigo, The Netherlands), 250–300 g (8–10 weeks old), were debrided of fibrofatty tissue and cut into rings of ~ 2 mm in width under sterile conditions. The aortic rings were transferred to a 48-well plate and starved in reprogramming media (M199, cat. 11,150,059, Gibco, USA, supplemented with 1% FBS) for 24 h. The following day, the rings were transferred to wells of 48-well plates pre-coated with 100 μL Geltrex™ LDEV-Free growth factor-reduced basement membrane matrix (Gibco, Life Technologies, USA) or the previously described LV dECM. Next, the rings were embedded with an additional 100 μL Geltrex™ or LV dECM and were incubated in basal medium with ASC-EVs (1 × 10^6^ EVs/ring/day) or with EVs-depleted conditioned medium (EDCM) for 10 days at 37 °C under 5% CO_2_. Rings cultured in the complete and basal medium were used as positive and negative controls, respectively. Media, with EVs or control, were replenished every other day. On days one, five, and ten, live-dead fluorescent staining was performed, and the tissue cultures were imaged as described below. Some aorta samples were lost during handling procedures and treatments.

### Vital staining (calcein-AM/PI)

Aorta ring cultures were washed three times with DPBS and dark incubated with the Live/Dead working solution composed of 5 µM Calcein-AM (Life Technologies®, Eugene, USA), 2 µM propidium iodide (PI; Sigma-Aldrich) and Hoechst nuclear dye (1:50, Thermo Fisher Scientific, Waltman, MA, USA) in serum-free medium at 37 °C, 5% CO_2_ for 30 min. The cultures were washed with DPBS and imaged with EVOS® M5000 digital inverted microscope (Electron Microscopy Sciences, Hatfield, USA) using the filters: GPF (λ_ex_ 470/22 nm/λ_em_ 525/50 nm), Texas Red (λ_ex_ 585/29 nm/λ_em_ 628/32 nm) and DAPI (λ_ex_ 357/44 nm/λ_em_ 447/60 nm) to visualise Calcein AM, PI, and Hoechst, respectively, at 4 × magnification. Images were analysed using ImageJ (https://imagej.nih.gov/ij/) with the Angiogenesis plug-in [18, 19] or AngioTool v.2 software. All images were transformed to 8-bit, and threshold and contrast were corrected to reduce hydrogel autofluorescence, facilitating particle segmentation and quantification. A background subtraction-based ROI extraction was performed (Suppl Fig. 1). To correct false positive angiogenesis results, day one measurements were labelled as zero and subtracted from the corresponding image measurements on days five and ten. Negative values were removed. This and manual errors resulted in some experimental groups having fewer repeats. Originally n = 6.

### RNA isolation, sequencing, and analysis

Total rna in cells or evs was extracted using a miRNeasy Isolation Kit (Qiagen; Venlo, Netherlands) according to the manufacturer’s instructions. The RNA concentration was determined with a NanoDrop 1000 spectrophotometer. The isolated RNA from ASC-EVS was analysed using an Agilent 2100 Bioanalyzer, an RNA 6000 Nano Kit, and an RNA 6000 ladder (Agilent Technologies; Santa Clara, CA) following the manufacturer’s protocol. The miR profiling of isolated total EVs-RNA was outsourced to Genohub Inc. (Austin, TX, USA) and performed as follows. RealSeq-Dual libraries were prepared using 10 µl of 1 ng/µl RNA input and 25 cycles of PCR. The libraries were pooled and then purified, and the size was selected using a Pippin Prep (Sage Biosciences). The library pool was profiled using a Tapestation and Qubit before sequencing on the NextSeq 550. Sequencing was performed with single 75 bp reads. Raw read sequences in FASTQ files were evaluated for quality control using FastQC (0.11.2) and filtered for low-quality and abundant short reads (less than 15 bp) (Suppl Fig. 2).

The raw fastq files were processed, adapter sequences were removed, and reads were filtered based on length. Reads shorter than 5 bp were filtered first to determine the RNA degradation. The 3’ adapters (with length alignment greater than 25) were trimmed with fastx_clipper (0.0.13.2) using Cutadapt. Reads with a minimum 15 bp length were aligned to a reference. Those filtered reads were mapped to the human reference genome (*Homo sapiens,* release hg19—UCSC Genome Bioinformatics) using the Bowtie2 mapping algorithm [20]. SAM files were post-processed to the BAM format using SAMtools. A file with read counts corresponding to annotated miRNAs (from miRBase) was generated with the BEDtools package [21]. Read counts were normalised and adjusted to a negative binomial model with an EdgeR-Bioconductor package to identify miRNAs highly enriched in the EVs fraction. We used TarPmiR, a random-forest-based approach trained with 13 features to predict the probability of a miRNA candidate target site. The CluePedia Cytoscape plugin was used to calculate the linear and non-linear statistical dependencies from experimental data. Genes, proteins, and miRNAs linked based on in silico and/or experimental information are integrated into a network with ClueGO terms/pathways. Gene/miRNA enrichments evaluated interrelations within pathways and associations. A pathway-like visualisation was created using the Cerebral plugin layout.

### Statistical analysis

Quantitative data were expressed as mean ± standard deviation (SD) based on at least three independent assays in duplicates or triplicates. EVs size, concentration, protein, and RNA concentrations were analysed using a two-tailed t-test. EVs uptake was analysed using one-way ANOVA and Tukey’s post hoc test. Analyses of tube formation and aortic ring sprouting assays were conducted using a mixed model. The nonparametric test Kruskal–Wallis test was used for multiple comparisons. For miR profiling, a Student’s t-test analysis was performed. All statistical analyses were performed using GraphPad Prism v9.1.0 (GraphPad Company, San Diego, USA). Graphs are presented as median with quartiles, mean ± SD or mean ± s.e.m. All *p-values* below 0.05 (*), 0.01 (**), and 0.001 (***) were considered statistically significant.

## Results

### Extracellular vesicles characterisation

**Fig. 2:**
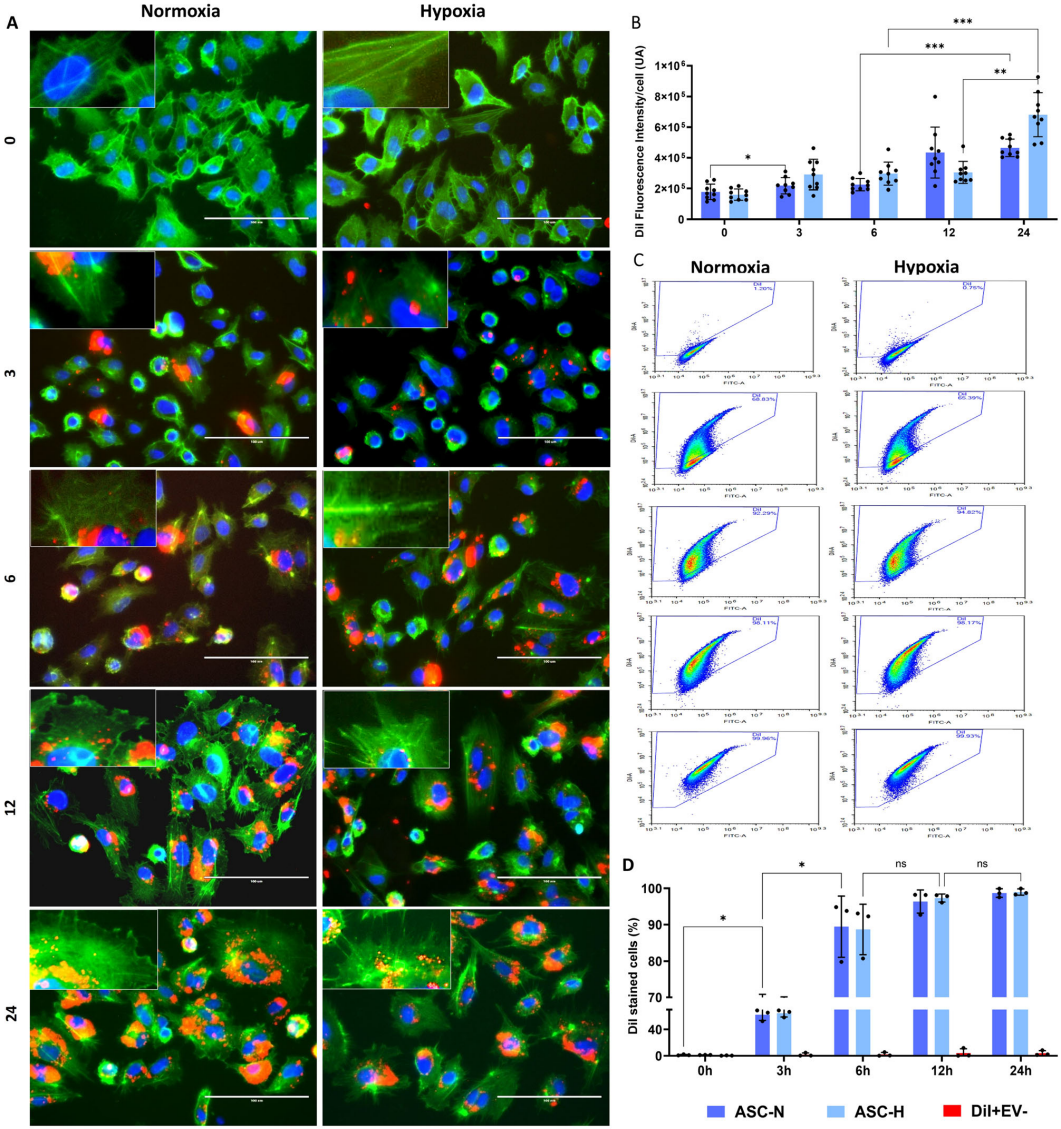
Kinetic of ASC-EVs uptake by HUVEC cells. ASC-derived EVs were pre-stained with the fluorescent lipophilic dye DiI and incubated with HUVECs in a time-lapse of 24 h. EVs internalisation was measured at 0, 3, 6, 12, and 24 h. Control samples (+ DiI, -EVs) were run in parallel and assayed in HUVEC cells to evaluate the background of DiI staining. Background fluorescence values for each time point were subtracted from the values of + DiI/ + EVs treated samples. **A** Fluorescence microscopy was used to visualise the DiI-EV uptake (red), cell morphology was assessed with phalloidin-Alexa Fluor 488 conjugated (green), and nuclei were stained with DAPI (blue). Scale bar 100 µm. **B** Fluorescence intensity quantification. Arbitrary units (U.A.). **C** FACS was used to measure how many HUVECs were positive for DiI. Over time, an increased amount of DiI-EVs is present in HUVECs, indicated by the upper displacement of the cells in the selected quadrant. **D** FACS quantification analysis. After three hours, almost 70% of HUVECs are already positive for DiI-ASC-EVs staining and after six hours, around 80%. After 12 h, virtually all cells are positive for DiI-ASC-EVs staining. Data were presented as mean ± SD, and error bars were calculated based on at least three independent experiments (n = 3) performed in triplicates. Two-way ANOVA, Tukey’s multiple comparison test compared to negative controls. * *p* < *0.05*; ***means *p* < *0.001,* ns: not significant. Each point in the graphs represents an individual sample

The nature of the ASC was previously confirmed by a Cluster of Differentiation (CD) marker panel (positive for CD29/90/105, negative for CD45) [16]. The ASC did not increase HIF-1*α* expression or translocation to nuclei after 24 h under hypoxia (Fig. 1B-C). However, an increased expression of HIF-1*α* was detected by WB in ASC (Fig. 1D). In addition, HIF-1*α* was below the detection limit in normoxic and hypoxic ASC-EVs (Fig. 2B). The ASC-EVs expressed markers like CD63, and CD81 as shown by WB. As expected, CD63 and CD81 were enriched in ASC-EVs compared to cell protein. Also, the enrichment was more prominent in hypoxia-derived ASC-EVs than in ASC-N EVs, either containing more EVs or containing EVs with higher CD63 and CD81 expression (Fig. 1D). Remarkably, CD9, another EV marker, was not expressed by ASC-EVs, irrespective of oxygen tension. The ASC-EVs were devoid of cytoplasmic markers like β-actin and thus considered pure (Fig. 1D). The mean diameters of ASC-N 117 ± 74 nm, and hypoxic 159 ± 74 nm, EVs did not differ (Fig. 1E), nor did their protein (ASC-N: 54.5 ± 15.3 µg/mL, ASC-H: 61.4 ± 8.8 µg/mL) or total RNA content (ASC-N: 8.5 ± 1.2 ng/µL, ASC-H: 9.3 ± 1.1 ng/µL) (Fig. 1G-H). However, hypoxia promoted EV production tenfold compared to normoxia (ASC-N: 7.41 × 10^10^ ± 1.89 × 10^10^ particles/mL, ASC-H: 2.18 × 10^11^ ± 5.46 × 10^10^ particles/mL) (Fig. 1F).

### Uptake

The ASC-EVs were fluorescently labelled with the lipophilic dye DiI. Fluorescence microscopy (FM) and flow cytometry (FC) were used to measure the DiI-ASC-EVs uptake by HUVECs over time (Fig. 2). Positive HUVECs for DiI-EVs were gated on according to unlabelled samples and stain controls (+ DiI, -EVs). By FM, a clear presence of EVs in HUVECs was observed as small round structures surrounding nuclei (Fig. 2A-B). From 6 h post-adsorption, a gradual increase in fluorescence intensity as an indicator of EV density/cell was observed. The maximum values were quantified between 12 and 24 h, characterised by bigger round and ellipsoid peri-nuclear structures (Fig. 2A-B). By FC, a DiI-EVs accumulation in HUVECs over time is shown by the upper displacement of the cells in the selected quadrant, related to increasing fluorescence intensity (Fig. 2C), which correlates with the FM results. The FC analyses showed that at 3 h post-adsorption, more than 60% of HUVECs had taken up the DiI-EVs (62% for ASC-N EVs, and 65% for ASC-H), n.s., (Fig. 2 C, D). After 6 h, nearly all HUVEC had taken up EVs, while this was 100% at 12 h and 24 h (Fig. 2 C, D). Hypoxia did not influence the uptake rate of ASC-EVs. As at 3 h post-adsorption, more than 50% of HUVECs contained EVs (Fig. 2), it stands to reason that EV-contained cargo would already be released inside the ECs and start exerting its effects early after uptake.

### ASC-derived EVs rescue the angiogenic capacity of growth factor-deprived ECs

**Fig. 3:**
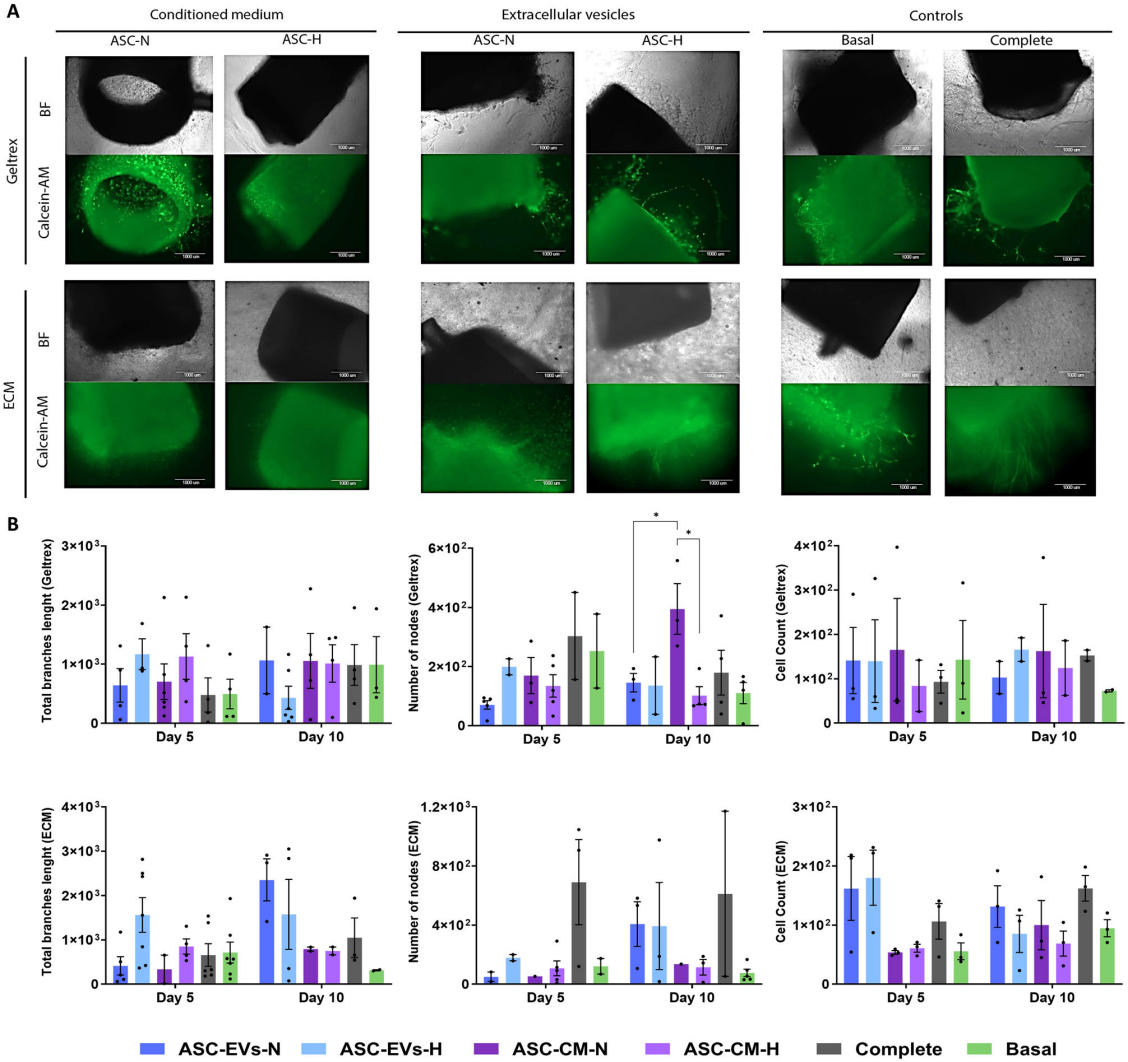
Ex vivo aorta ring sprouting assay in Geltrex and dECM at day 10. **A** Bright-field and GFP channel (stained with Calcein-AM), scale 400 nm. **B** Angiogenesis parameters quantification from ex vivo aortic rings in ECM and Geltrex. Results represent the total branches length, number of nodes, and number of cells. All results represent at least three independent assays in triplicate, and each value is the mean ± SEM. of the determinations *p ≤ *0.05.* ASC, adipose stromal cells; BF, bright field; CM, conditioned medium; dECM, decellularized extracellular matrix; EVs, extracellular vesicles; H, hypoxia; N, normoxia. Each point in the graphs represents an individual sample

The pro-angiogenic activity of EVs was assessed by aortic ring assay (Fig. 3A, Supp Fig. 1). In the LV dECM hydrogel, the ASC-CM-N (p = 0.0064) and ASC-CM H (p = 0.0015) showed an increase in cell numbers from day one to day five. Cell numbers remained similar between days five and ten. In Geltrex, the ASC-CM-N (p = 0.0041) and ASC-N (p = 0.0345) increased in cell numbers from day one to day five, which did not increase further afterward (Fig. 3B).

Angiogenesis was determined by total branches length measurements (BL) and the number of nodes (NN). Overall, in LV dECM hydrogel, the highest BL was observed in the ASC-N group on day ten. Cells treated with ASC-CM, both N and H, ASC-H and basal media tended to decrease from day five to day ten. After day five, BL did not change in any group. On day ten, all groups showed higher BL than the negative control (basal). Rings cultured in Geltrex showed the highest BL in the ASC-CM-H group on day five and in the ASC-CM-N group on day ten. All groups except ASC-H showed higher readings than the negative control (basal) at day ten (Fig. 3B).

In LV dECM, the highest NN, besides the positive control (complete medium), was observed in ASC-N at day ten (Fig. 3B). All groups showed an increase in the NN compared to the negative control (basal) at day ten. In Geltrex, ASC-CM-N (day ten) showed the highest NN, with a significant increase compared to ASC-N, day five (p = 0.010) and ASC-CM-H, day ten (p = 0.0398) (Fig. 3B). All groups, except ASC-CM-H, had increased NN compared to basal medium at day ten.

### Identification and putative function analysis of differentially expressed miRNAs

**Fig. 4:**
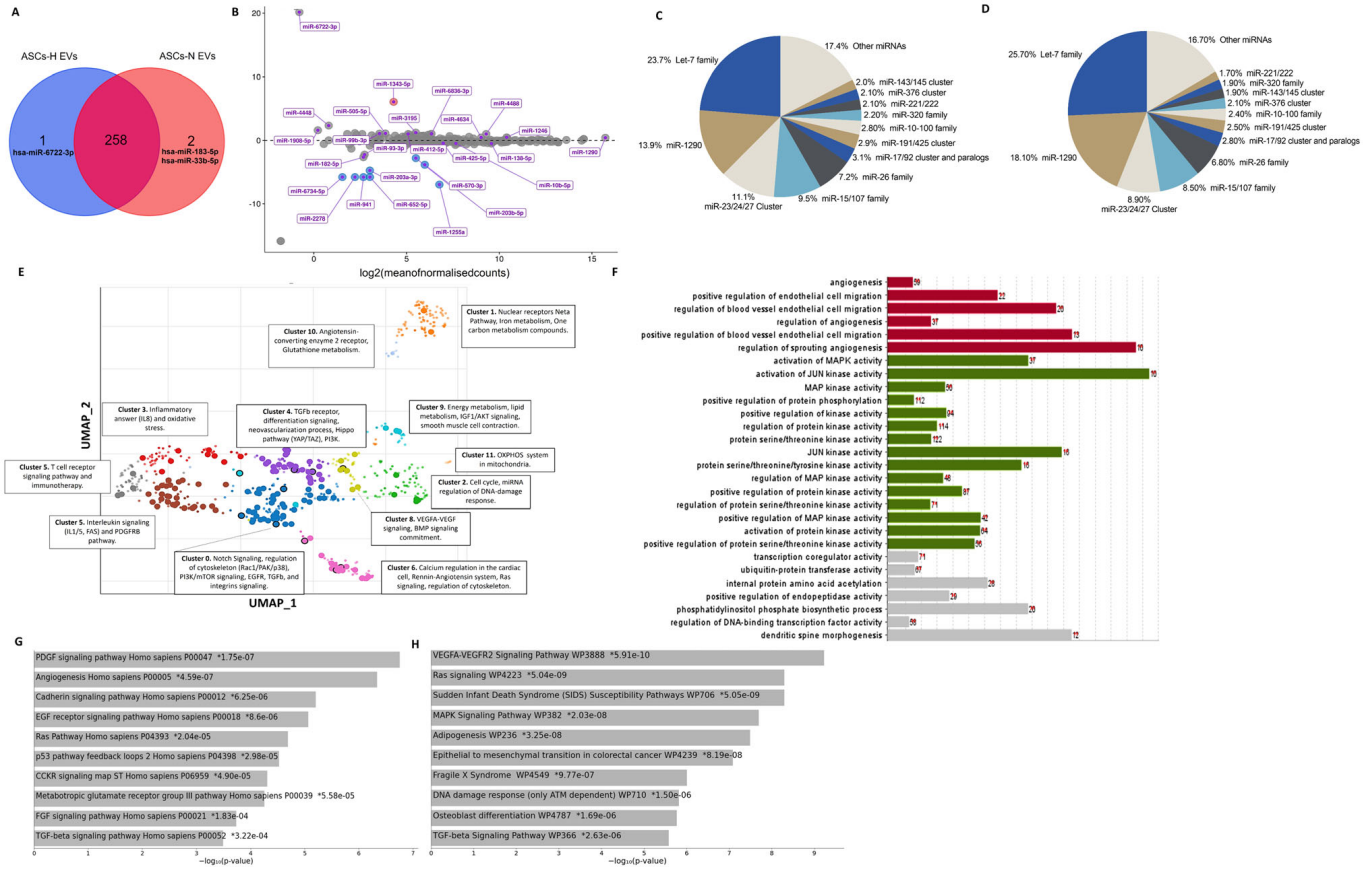
miRNA expression and predicted target analyses in ASCs-EVs. **A** Venn diagram of total miRNA found in ASC-EVs from normoxia and hypoxia. Of the 260 miRNAs identified, 258 are shared independent of the oxygen tension. **B** MA plot showing the changes in the levels of miRNA expression in ASC-EVs from cells cultured in hypoxia compared to their normoxia counterparts. The miRNA family/cluster enrichment in ASC-EVS for cells in **C** normoxia and **D** hypoxia. Ten families represent more than 60% of the total miRNA mapped reads. This added to miR-1290, represents almost 80% of all the information concerning miRNAs in these EVs. **E** Scatterplot of the miRNA-mRNA targets with all terms in the GO_Cellular_Component_2023 gene set library, using the enrichment analysis visualization Appyter to visualize Enrichr results. Each point represents a term in the library. Term frequency-inverse document frequency (TF-IDF) values were computed for the target set corresponding to each term, and UMAP was applied to the resulting values. The terms are plotted based on the first two UMAP dimensions. Terms with more similar gene sets are positioned closer together. Terms are colored by automatically identified clusters computed with the Leiden algorithm applied to the TF-IDF values. **F** Predicted and validated targets of the top_60 enriched ASC-EV-derived miRNA using the miRWalk algorithm were used to conduct analyses. (Complete list of nominally significant genes, miRNA binding sites, and miRNA and mRNA (*p-value* < 0.05) for each miRNA see Supplementary data 1). Gene Ontology, biological process positively enriched (FDR for each gene list was controlled using right-sided hypergeometric test, Benjamin Hochberg Correction) *p* = *0.05, q* < *0.2.* Bar charts of top enriched terms from the Panther (**G**) and WikiPathway (**H**) gene set libraries. The top 10 enriched terms for the input gene set are displayed based on the -log10 (*p-value*). The term at the top has the most significant overlap with the input query gene set

High-throughput sequencing was employed to investigate the expression of miRNAs in ASC-EVS; the sequencing dataset profile summary is detailed in (Suppl Table 1) (complete sequence data available on request). To identify the miRNA cargo of ASC-EVs, the small RNA clean reads of the libraries were matched with known miRNA precursors and mature miRNAs from miRBase [22, 23]. Normoxic and hypoxic ASC- EVs comprised 260 and 259 distinct miRNAs respectively (Fig. 4A). Two miRNAs (hsa-miR-183-5p and hsa-miR-33b-5p) were unique to ASC-EV-N while one miRNA (hsa-miR-6722-3p) was unique to ASC-EV-H (Fig. 4A). The remaining 258 (98.8%) miRNAs overlapped. The miRNA data was further analysed into differentially expressed (DE) and enriched miRNAs in ASC-derived EVs. After normalisation and DE analysis, ASC-H EVs had eleven upregulated miRNAs and eight downregulated miRNAs compared to ASC-H EVs (Suppl Fig. 3, Suppl Table 2).

The DE miRNAs in normoxia and hypoxia derived-ASC-EVs were identified. Yet, the vast majority (95%) of these were below the already low threshold that was set at 100 transcripts per million (TPM) (Fig. 4A, Suppl Table 2, Suppl data 1 Tabulated counts_normalized_100 ASC-EVs). Likely, these DE miRNAs hold no biological relevance. At the same time, the sequenced RNA was obtained from approx. 5 × 10^10^ EVs, this would equate to approx. 2 × 10^−7^ copies of each miRNA per EV (Fig. 4B). In other words, no more than 1 per 10 million EVs would contain such a specific miRNA. Therefore, the ASC-EVs miRNA sequencing data were ranked according to enriched miRNAs and further analysed. The top 60 enriched miRNAs (Suppl Table 3) represented more than 90% of all reads (Table 1), which comprise approximately ten families or clusters of miRNAs and only one non-clustered miRNA, miR-1290 (Fig. 4C-D).

**Table 1:** The top 60 miRNAs enriched in ASCs derived-EVs are composed of clustered/Families miRNAs

Family/Cluster	% Total reads	*Highly Expressed	**Low expressed	Absent
Let-7 Family	~ 23–26%	Let-7a, let-7b, let-7c, let-7d, let-7e, let-7f, let-7 g, let-7i	miR-98	miR-202
miR-1290	~ 14–18%	Non-clustered miRNA		
miR-23–27-24 cluster	~ 9–11%	miR-27 a-3p, miR-27b-3p, miR-23 a-3p, miR-23b-3p, gene always clustered on chromosomes with miR-23 and miR-24 genes	miR-27 a-5p, miR-24-3p, miR-23c, miR-3074 is an antisense miRNA for miR-24, on Chr 9, also clustered in the miR-23b gene cluster	
miR-15/107	~ 9%	miR-15a/b, miR-16, miR-107, miR-103 a/b		miR-195
miR-26 Family	~ 7%	Chr 3: miR-26a-1, Chr 12: miR-26a-2	Chr 2: miR-26b	
miR-17 ~ 92 cluster and paralogs	~ 3%	Chr13: miR-17, miR-18a, miR-20a, miR-19b-1, and miR-92a-1		miR-19a
		Chr X: miR-18b, miR-20b, miR-92a-2, miR-106a, miR-363-3p, miR-363-5p		
		Chr 7: miR-106b, miR-93 and miR-25		miR-19b
miR-106a ~ 363 and miR-106b/25				
miR-191/425	~ 3%	Chr 3: miR-191-5p	miR-425-5p, miR-425-3p	miR-191-3p
miR-10/100 Family	~ 2.8%	They are found within de Hox gene clusters. miR-10a, miR-10b, miR-99a, and miR-99b, located on Chr 17, Chr 2, Chr 21, and Chr 19, respectively. Chr 11: miR-100, miR-125b		
miR-320	~ 2.5%	Five members: miR-320a-e. Chr 8: miR-320a, Chr 1: miR-320b1/-2	Chr 18: miR-320c1/-2	miR-320e
			miR-320-d	
miR-221/22 cluster	~ 2%	miR-221 and miR-222 are found on the X chromosome and are expressed from a single transcript		
miR-376 cluster	~ 2%	miR-376c, miR-376b, miR-376a-3p	miR-376a and -b	mir-368
miR-143/145	~ 1.8%	miR-143 and miR-145 form a bicistronic cluster in 5q33.1	miR-143-5p, ir-145-3p	
		mir-143-3p and mir-145-5p		
		*Non-clustered/family miRNAs highly expressed in EVs, additional to 1290: miR-22/335/185/122/, miR-423, and the complementary miR-3184		

*Family/Cluster Members present in the top 60. ** *Family/Cluster Members out of the top 60

### Predicted impact of ASC-EV-derived miRNAs on cellular processes

The enrichment pathway analyses with the miRNA expression correlations and predicted targets was made by matching the top-60 enriched miRNAs in ASC-EVs with predicted mRNA targets in miRWalk (Suppl data 2, complete list of nominally significant genes, p-value < 0.05 for each miRNA) and subsequent GO enrichment analyses showed a preponderance for mRNAs affected by miR-1290, miR23/27/24 cluster and the miR-15/107 family with no less than 15,870 mRNAs to be targeted by those enriched ASC-EVs-derived miRNAs. (Fig. 4E,F, Suppl data 2 miRWalk miRNA-mRNA target analyses). Most notable was that angiogenesis-related processes also showed the highest relevance in Phanter and WikiPathway analysis i.e., PDGF, VEGF/VEGFR, EGF, FGF and TGF-β signalling (Fig. 4G,H). This included terms such as motility, cell migration, actin cytoskeleton regulation, EC activation-related metabolic switch and angiogenic cellular patterning (Suppl data 3 and Suppl data 4).

## Discussion

In this study, we showed that ASCs-EV support cell viability and ex vivo sprouting angiogenesis of arteries cultured without flow and in nutrient-deficient conditions, which are characteristics of injured vessels. Hypoxic preconditioning of ASC did not further increase the uptake rate or the angiogenic capacity of normoxic ASCs-EV. Fine-tuned with the above, no more than marginal changes in the EV miRNA cargo were observed, overlapping 98.8% between ASC-EV-N and ASC-EV-H. Pertaining to angiogenesis, the most abundantly expressed miRNAs in EVs are miR-1290, the miR23/27/24 cluster, and the miR-15/107 family (Table 2).

**Table 2:** The top 20 significant processes enriched by gene targets from cervical carcinoma cells-EVs derived miRNAs

Category	B-H *p*-value
Cellular growth and proliferation	1.27E-28–1.11E-03
Cancer	4.54E-24–1.01E-03
Organismal injury and abnormalities	4.54E-24–1.11E-03
Cell Death and survival	5.07E-24–1.01E-03
Organismal survival	5.07E-24–2.65E-06
Cell morphology	8.22E-19–7.27E-04
Cellular assembly and organization	7.61E-20–4.99E-04
Cellular function and maintenance	7.61E-20–8.07E-04
Cellular development	4.18E-15–1.09E-03
Neurological disease	3.21E-13–1.11E-03
Cellular movement	2.28E-15–1.05E-03
Tissue development	1.82E-05–5.83E-02
Organ morphology	5.23E-05–6.03E-02
Gene expression	3.57E-13–3.95E-04
Cardiovascular system development and function	3.57E-13–1.13E-03
Organismal development	4.85E-13–1.01E-03
Gastrointestinal disease	1.43E-12–8.91E-04
Infectious disease	1.63E-12–6.47E-04
Nervous system development and function	3.74E-12–1.13E-03
Hereditary disorder	8.81E-12–8.81E-12
Psychological disorders	8.81E-12–7.60E-11

Normalized enrichment score; GO: gene ontology; KEGG: Kyoto encyclopedia of genes and genomes; #: Genes sets enriched among genes higher expresse

Administration of MSC like ASC for pro-angiogenic therapy is in experimental clinical use to treat the aftermath of compromised perfusion, including myocardial infarction [24], stroke [25], and peripheral arterial disease [26]. The additional benefit of using MSC, like ASC, is that it augments several regenerative processes while blocking or even reversing fibrosis [27–30]. Most of the beneficial effects of MSC are elicited through secretome factors like growth factors, yet EVs' therapeutic impact has been intensely interrogated to date [31]. The secretome of ASC is easily harvested as a conditioned (culture) medium, both for therapy and analyses. The reductionist’s view that single secretome factors are responsible for the therapeutic benefit is naïve. The genomic data of the ASC secretome reveal 100 to more than 1000 diverse proteins [32–34]. This diversity of numbers of reported proteins results from different techniques of sample preparation and analysis of the data, resulting in problems in comparing the heterogeneously reported data [35]. The EVs are yet another complex biological factor that contributes to the ASC paracrine signaling. They complement soluble proteinaceous secretome factors in that EVs elicit their effects inside target cells upon entry. Our results show that targeted endothelial cells, i.e., HUVECs, are readily accessible to EVs while uptake was maximal within 12 h. To translate this to a clinical situation in which perfusion is compromised is challenging. Yet, our results also show that upon mixing with extracellular matrix hydrogels, EVs elicit their proangiogenic effect on aortic rings within days of culture. That means EVs remain stable in a tissue-mimicking microenvironment and at body temperature. As it appeared, hypoxia did increase EV production by EVs but did not alter the miRNA profile of EVs. This is likely due to the already inherently high proangiogenic miRNA profile of ASC-derived EVs.

Our results contradict studies that indicate that hypoxic pre-conditioning of ASC increases their proangiogenic capacity [11, 36]. On the other hand, our results corroborate others that detected no changes in secretome by proteomics after hypoxic preconditioning [34, 37]. Not all cell lineages exhibit uniform susceptibility to oxygen tension, ranging from those with minimal sensitivity to those showing constitutive expression of HIF1a, often due to genetic alterations, as observed in highly modified cancer cells [38, 40]. Furthermore, it's essential to note that studies may adopt varying rationales for treatment implementation. While our approach involves specific EV concentrations (independent of the fact that hypoxia increases EV secretion), other studies may base dosages on protein or other molecular concentrations [41]. Given the heterogeneous nature of molecule/particle concentrations, variations in treatment doses across studies could potentially overstate the effects of hypoxia.

The stoichiometric analyses of miRNA copy numbers versus EV numbers revealed that, on average, even the most abundant miRNAs are below one copy per EV. In fact, on a mathematical basis, most likely, not even all EVs carry miRNAs. In fact, at present, it is known that miRNAs are distributed heterogeneously over EVs, meaning that the miRNA composition differs for individual EVs [42, 43]. This, of course, does not preclude these from carrying proteins. This has ramifications for in vitro experiments and even for future clinical use.

In our study, we determined the experimental doses on a fixed number of EVs per cell/tissue. Considering the limitation that due to the mentioned heterogeneous packaging of miRNAs in EVs, higher variation in reads could occur, instead of assessing individual miRNAs, we biased the analyses onto families and clusters. Since the latter frequently share seed sequences and target-related proteins as multiple members of (signalling) pathways to regulate and fine-tune protein expression [44, 45]. While the expression of individual miRNAs was oxygen-independent and overlapped almost 99%, more than 50% of the total reads comprised members of Let-7, miR23/27/24, miR-15/107, miR-26, and miR17/92 and paralog clusters. Interestingly, a single non-clustered miRNA (miR-1290) stood out with approximately 16% of all sequence reads, second only to the Let-7 family with approximately 25% of total reads (with 9 different miRNAs detected). This indicates that the effect of miR-1290 might surpass the influence of other EV-derived miRNAs. Cellular [46, 47] and EV-derived [48, 49] miR-1290 drives tumour progression by enhancing proliferation, migration, and angiogenesis. While these processes contribute to poor patient prognosis in cancer, these would be advantageous in tissue regeneration, rendering miR-1290 a promising candidate for therapy [50]. Moreover, EV-derived miR-1290 from endothelial precursor cells promotes fibroblast-to-EC transition, contributing to wound healing [51].

Implementing a 3D culture scaffold to deliver EVs to cells or tissue cultures provides an extended in vitro model that better represents and connects to future in vivo experiments and applications. The gold standard for 3D cell cultures and vascularisation experiments is a basement membrane-derived hydrogel such as Matrigel, Geltrex or Celltrex. Yet, these hydrogels are tumour-derived and do not resemble organ-specific ECM. Our results showed as proof of concept that angiogenic sprouting of aorta rings occurred in LV ECM and that it supported EV-mediated sprouting stimulation. LV ECM showed a higher variation than Geltrex, which is attributed to the technical challenges of visualising structures in the opaque LV hydrogel compared to the clearer Geltrex. Geltrex comprises 60% laminin, 30% collagen IV, 8% entactin, and 3% HSPG [52] but no collagen type I. In comparison, organ-derived ECM hydrogels are more complex and contain approx 80% collagen type I and hundreds of other proteins [53] that are often organ-specific, such as elastin, which is enriched in arteries. LV ECM comprises 64 highly expressed proteins, but about 14% are hundreds of lower expressed proteins [53]. Organ-derived hydrogels tend to be stiffer than Geltrex, although this difference may not contribute to phenotypic changes [53, 54].

In conclusion, ASC-EVs are a promising cell-free therapy strategy, but many unknowns remain. Our study found no substantial differences in the miRNA expression profile between normoxic and hypoxic ASC-EVs or in the functional effect of those EVs since both normoxic and hypoxic-derived EVs support angiogenesis similarly.

### Supplementary Information

Below is the link to the electronic supplementary material.

Supplementary file1 (XLSX 37 KB)

Supplementary file2 (XLSX 32831 KB)

Supplementary file3 (XLSX 13 KB)

Supplementary file4 (XLSX 17 KB)

Supplementary file5 (DOCX 830 KB)

## Data Availability

Sequencing data and generated data sets are available to the corresponding author upon request.
