# Supplementary material for: Extracellular Vesicles from Adipose Tissue-Derived Stromal Cells Stimulate Angiogenesis in a Scaffold-Dependent Fashion
Source: Tissue Eng Regen Med. 2024 Jul 8;21(6):881–95. doi: 10.1007/s13770-024-00650-4 (PMC11286612; doi:10.1007/s13770-024-00650-4)
Supplement: Supplementary file 5 — Supplementary file5 (DOCX 830 KB) [file 13770_2024_650_MOESM5_ESM.docx]

**Supplementary Material.**


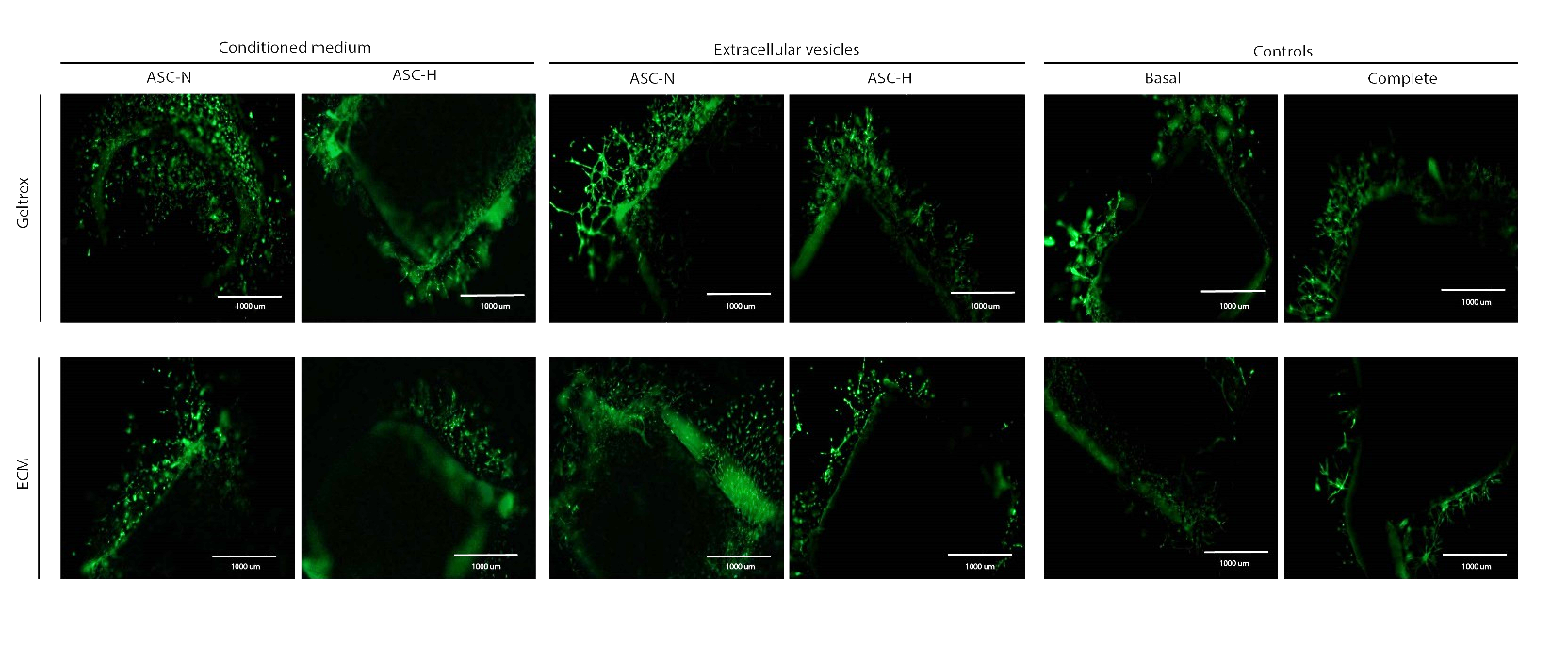


**Supplementary Figure 1:** Aortic sprouting. Compared to Figure 3, brightness was increased while background was removed, essentially obscuring the (central) aorta from the images, to emphasize the vascular sprouts. Staining with calcein AM, GFP channel, 4x magnification.


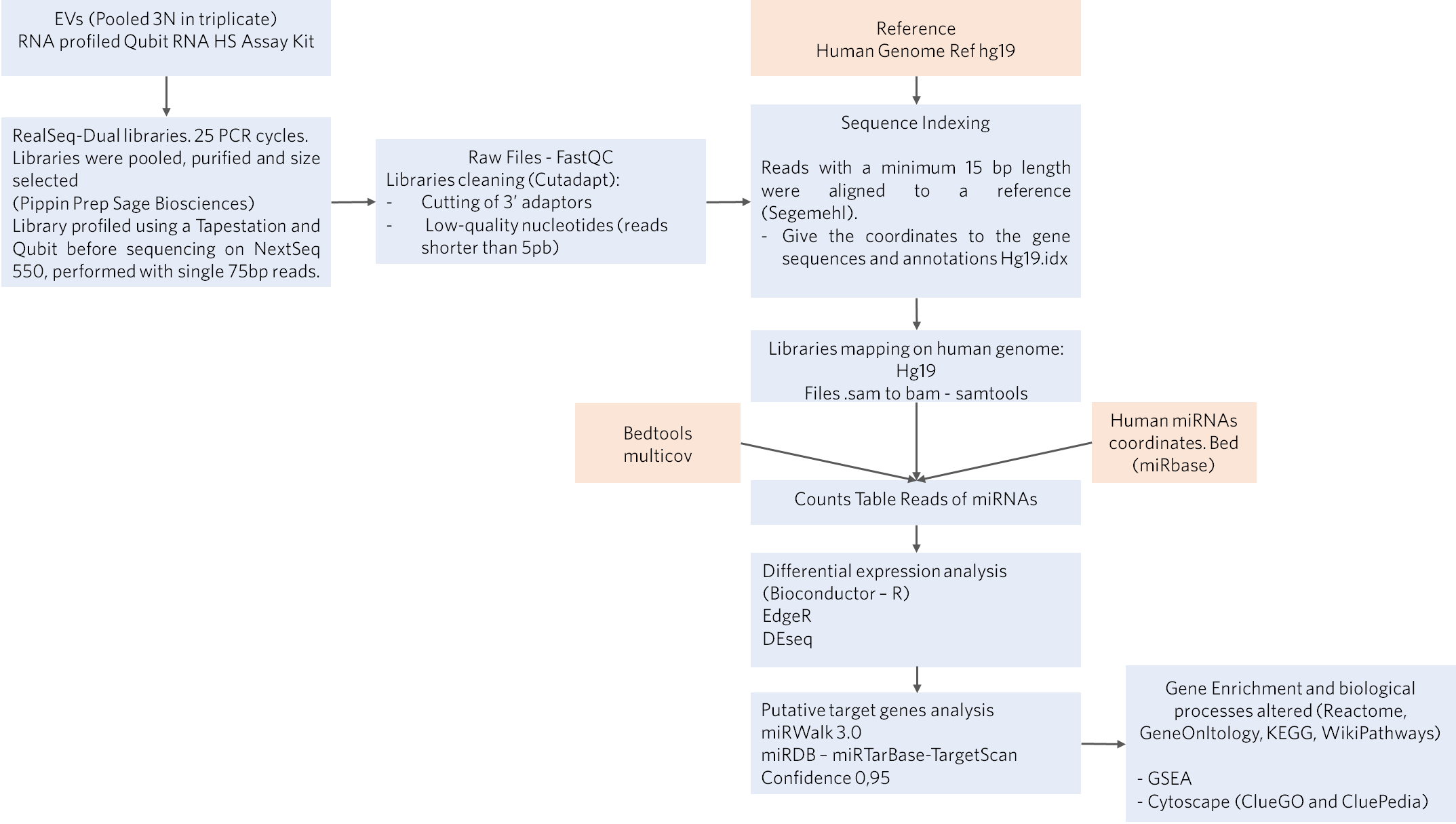


**Supplementary Figure 2. Sequencing and data analysis procedure layout**

**Supplementary Table 1.** Average data set summary of sequencing profile in ASC-EV samples. The commands used for adapter trimming were Trim5 (cutadapt -j 6 --nextseq-trim=15 -u 1 -a TGGAATTCTCGGGTGCCAAGG -m 5) and Trim15 (cutadapt -j 6 --nextseq-trim=15 -u 1 -a TGGAATTCTCGGGTGCCAAGG -m 15). The commands used for the alignments were miRbase (bowtie -S --chunkmbs 512 -p 4 -n 1 -I 17 -a -q -m 25 -e 2000 --best --strata) and hg19 (bowtie -S --chunkmbs 512 -p 6 -n 1 -I 19 -m 25 --best --strata -e 2000). Extra data may be available upon request.

| **EVs Sample** | **Raw**  **Reads** | **Trimmed Reds (minimum length 5)** | **Percentage Reds (minimum length 5)** | **Trimmed Reds (minimum length 15)** | **Percentage Reds (minimum length 15)** | **# Reads aligned to human genome** | **% Reads aligned to human genome** | **# Reads aligned to hsa miRBase** | **% Reads aligned to hsa miRBase** |
| --- | --- | --- | --- | --- | --- | --- | --- | --- | --- |
| ASCs-H | 57891767 | 55883669 | 96.5 % | 52931730 | 91.4 % | 49845413 | 94.17 % | 484856 | 0.92 % |
| ASCs-N | 63500040 | 62012798 | 97.7 % | 59901767 | 94.3 % | 56053119 | 93.58 % | 472331 | 0.79 % |


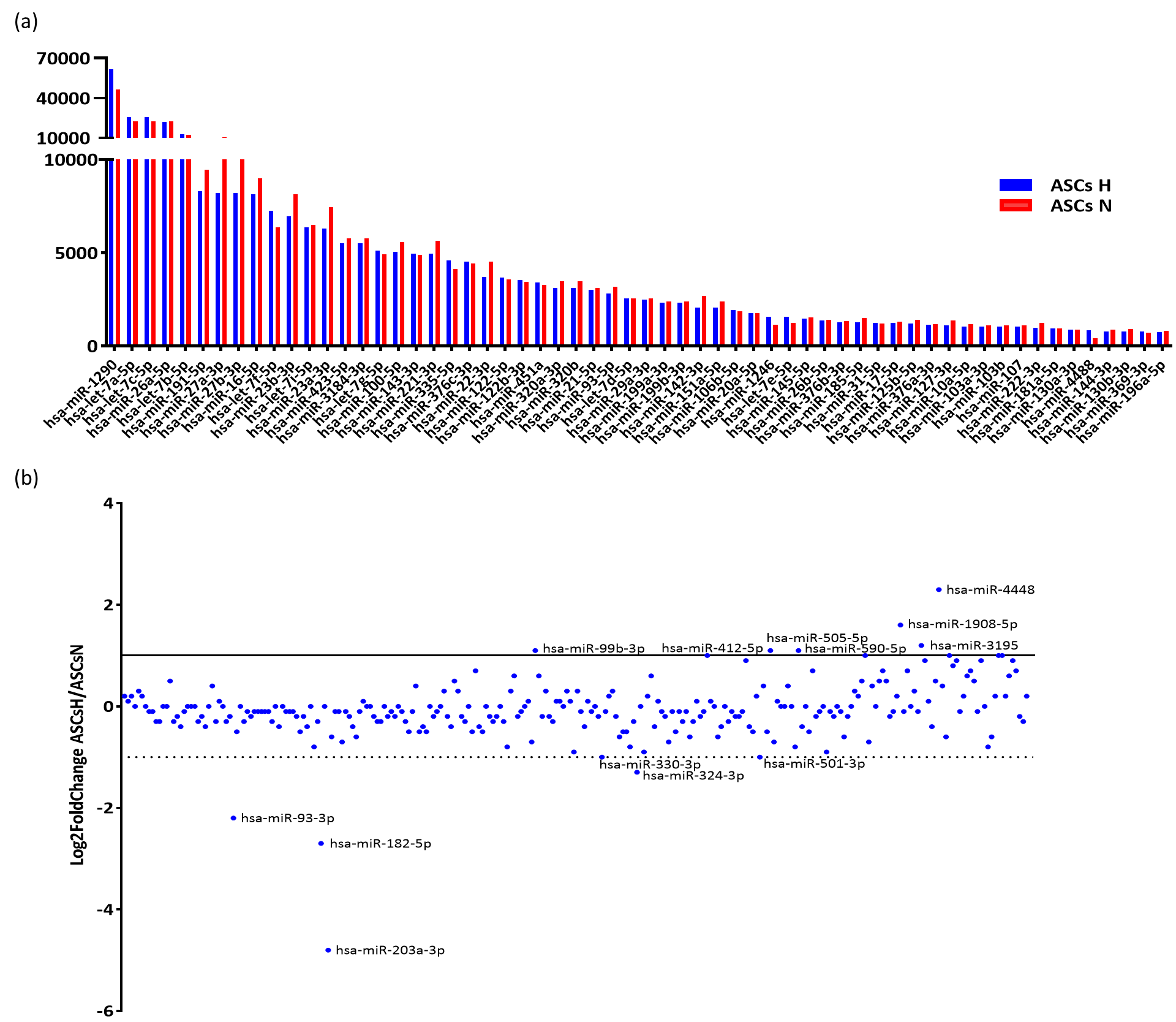


**Supplementary Figure 3.** (a) Graphic representation of the top 60 enriched miRNAs. (b) miRNA differentially expressed. We found 11 miRNAs to be upregulated and 8 to be downregulated. When we observed the number of reads of these miRNAs, we found that most have a low number of reads, making this differential expression depreciable regarding biological relevance. All values are below the average reads number (Supplementary Table 2).

**Supplementary Table 2. Differentially expressed miRNAs in ASC-derived Evs.** Upregulated and downregulated miRNAs in ASC-EVs. We found 11 miRNAs to be upregulated and 8 to be downregulated. When we observed the number of reads of these miRNAs we found that most of those miRNAs have a low number of reads, making this differential expression depreciable in terms of biological relevance. Normalized count number_100 of miRNAs in ASC-H and ASC-H EVs, fold change (FC) number, and Log2FC for every miRNA.

|  | **miRNA** | **ASC H** | **ASC N** | **FC** | **Log2FC** |
| --- | --- | --- | --- | --- | --- |
| ***Upregulated*** | hsa-miR-4488 | 839,4 | 419,2 | 2,0 | 1,0 |
|  | hsa-miR-6836-3p | 109,3 | 53,3 | 2,0 | 1,0 |
|  | hsa-miR-3195 | 63,4 | 27,3 | 2,3 | 1,2 |
|  | hsa-miR-412-5p | 45,3 | 22,6 | 2,0 | 1,0 |
|  | hsa-miR-505-5p | 19,8 | 9,3 | 2,1 | 1,1 |
|  | hsa-miR-99b-3p | 15.7 | 7,5 | 2,1 | 1,1 |
|  | hsa-miR-6724-5p | 8,1 | 4,1 | 2,0 | 1,0 |
|  | hsa-miR-590-5p | 6,4 | 2,9 | 2,2 | 1,1 |
|  | hsa-miR-940 | 4,7 | 2,3 | 2,0 | 1,0 |
|  | hsa-miR-4448 | 2,9 | 0,6 | 5,0 | 2,3 |
|  | hsa-miR-1908-5p | 1,7 | 0,6 | 3,0 | 1,6 |
| ***Downregulated*** | hsa-miR-671-5p | 72,7 | 136,3 | 0,5 | -0,9 |
|  | hsa-miR-370-3p | 20,9 | 39,4 | 0,5 | -0,9 |
|  | hsa-miR-330-3p | 16,9 | 34,2 | 0,5 | -1,0 |
|  | hsa-miR-501-3p | 12,8 | 25,5 | 0,5 | -1,0 |
|  | hsa-miR-324-3p | 2,3 | 5,8 | 0,4 | -1,3 |
|  | hsa-miR-93-3p | 2,3 | 11,0 | 0,2 | -2,2 |
|  | hsa-miR-182-5p | 1,7 | 11,0 | 0,2 | -2,7 |
|  | hsa-miR-203a-3p | 0,6 | 15,7 | 0,0 | -4,8 |

**Supplementary Table 3**. The average normalized reads from the top 60 enriched miRNA in ASC-derived EVs.

| **ASCs Normoxia** | |  | | **ASCs Hypoxia** | | |
| --- | --- | --- | --- | --- | --- | --- |
| **miRNA** | **Normalized counts** | |  | | **miRNA** | **Normalized counts** |
| hsa-miR-1290 | 46570 | |  | | hsa-miR-1290 | 61731 |
| hsa-miR-26a-5p | 22621 | |  | | hsa-let-7a-5p | 25869 |
| hsa-let-7a-5p | 22343 | |  | | hsa-let-7c-5p | 25810 |
| hsa-let-7c-5p | 22310 | |  | | hsa-miR-26a-5p | 21958 |
| hsa-let-7b-5p | 12342 | |  | | hsa-let-7b-5p | 12853 |
| hsa-miR-27a-3p | 10367 | |  | | hsa-miR-191-5p | 8308 |
| hsa-miR-27b-3p | 10358 | |  | | hsa-miR-27a-3p | 8211 |
| hsa-miR-191-5p | 9443 | |  | | hsa-miR-27b-3p | 8200 |
| hsa-miR-16-5p | 9005 | |  | | hsa-miR-16-5p | 8142 |
| hsa-miR-23b-3p | 8141 | |  | | hsa-let-7f-5p | 7233 |
| hsa-miR-23a-3p | 7441 | |  | | hsa-miR-23b-3p | 6970 |
| hsa-let-7i-5p | 6505 | |  | | hsa-let-7i-5p | 6346 |
| hsa-let-7f-5p | 6359 | |  | | hsa-miR-23a-3p | 6311 |
| hsa-miR-423-5p | 5772 | |  | | hsa-miR-423-5p | 5502 |
| hsa-miR-3184-3p | 5772 | |  | | hsa-miR-3184-3p | 5502 |
| hsa-miR-221-3p | 5629 | |  | | hsa-let-7g-5p | 5110 |
| hsa-miR-100-5p | 5577 | |  | | hsa-miR-100-5p | 5054 |
| hsa-let-7g-5p | 4920 | |  | | hsa-miR-143-3p | 4951 |
| hsa-miR-143-3p | 4878 | |  | | hsa-miR-221-3p | 4937 |
| hsa-miR-22-3p | 4538 | |  | | hsa-miR-335-5p | 4581 |
| hsa-miR-376c-3p | 4424 | |  | | hsa-miR-376c-3p | 4514 |
| hsa-miR-335-5p | 4120 | |  | | hsa-miR-22-3p | 3717 |
| hsa-miR-122-5p | 3572 | |  | | hsa-miR-122-5p | 3663 |
| hsa-miR-320a-3p | 3457 | |  | | hsa-miR-122b-3p | 3537 |
| hsa-miR-320b | 3457 | |  | | hsa-miR-451a | 3412 |
| hsa-miR-122b-3p | 3424 | |  | | hsa-miR-320a-3p | 3110 |
| hsa-miR-451a | 3278 | |  | | hsa-miR-320b | 3108 |
| hsa-miR-93-5p | 3186 | |  | | hsa-miR-21-5p | 3003 |
| hsa-miR-21-5p | 3108 | |  | | hsa-miR-93-5p | 2823 |
| hsa-miR-142-3p | 2681 | |  | | hsa-let-7d-5p | 2536 |
| hsa-let-7d-5p | 2552 | |  | | hsa-miR-29a-3p | 2473 |
| hsa-miR-29a-3p | 2537 | |  | | hsa-miR-199a-3p | 2311 |
| hsa-miR-151a-5p | 2377 | |  | | hsa-miR-199b-3p | 2311 |
| hsa-miR-199a-3p | 2374 | |  | | hsa-miR-142-3p | 2065 |
| hsa-miR-199b-3p | 2374 | |  | | hsa-miR-151a-5p | 2049 |
| hsa-miR-106b-5p | 1854 | |  | | hsa-miR-106b-5p | 1915 |
| hsa-miR-20a-5p | 1773 | |  | | hsa-miR-20a-5p | 1771 |
| hsa-miR-145-5p | 1539 | |  | | hsa-miR-1246 | 1574 |
| hsa-miR-185-5p | 1507 | |  | | hsa-let-7e-5p | 1545 |
| hsa-miR-26b-5p | 1396 | |  | | hsa-miR-145-5p | 1472 |
| hsa-miR-125b-5p | 1389 | |  | | hsa-miR-26b-5p | 1371 |
| hsa-miR-127-3p | 1376 | |  | | hsa-miR-376b-3p | 1281 |
| hsa-miR-376b-3p | 1316 | |  | | hsa-miR-185-5p | 1249 |
| hsa-miR-17-5p | 1293 | |  | | hsa-miR-31-5p | 1241 |
| hsa-miR-222-3p | 1247 | |  | | hsa-miR-17-5p | 1223 |
| hsa-let-7e-5p | 1230 | |  | | hsa-miR-125b-5p | 1197 |
| hsa-miR-31-5p | 1193 | |  | | hsa-miR-376a-3p | 1141 |
| hsa-miR-376a-3p | 1184 | |  | | hsa-miR-127-3p | 1107 |
| hsa-miR-10a-5p | 1174 | |  | | hsa-miR-10a-5p | 1040 |
| hsa-miR-1246 | 1132 | |  | | hsa-miR-103a-3p | 1038 |
| hsa-miR-103a-3p | 1092 | |  | | hsa-miR-103b | 1038 |
| hsa-miR-103b | 1092 | |  | | hsa-miR-107 | 1037 |
| hsa-miR-107 | 1091 | |  | | hsa-miR-222-3p | 967 |
| hsa-miR-181a-5p | 949 | |  | | hsa-miR-181a-5p | 942 |
| hsa-miR-138-5p | 897 | |  | | hsa-miR-130a-3p | 854 |
| hsa-miR-130b-3p | 891 | |  | | hsa-miR-4488 | 839 |
| hsa-miR-130a-3p | 884 | |  | | hsa-miR-144-3p | 775 |
| hsa-miR-144-3p | 871 | |  | | hsa-miR-130b-3p | 773 |
| hsa-miR-6529-5p | 858 | |  | | hsa-miR-369-3p | 761 |
| hsa-miR-196a-5p | 818 | |  | | hsa-miR-196a-5p | 740 |
| Total ASC-N= 334296.07 _100 normalized counts, top 60= 308666.89 92% of total reads  Total ASC-H= 341591.10 _100 normalized counts, top 60= 317811.38 93% of total reads | | | | | | |
